# ACSL4-dependent ferroptosis does not represent a tumor-suppressive mechanism but ACSL4 rather promotes liver cancer progression

**DOI:** 10.1038/s41419-022-05137-5

**Published:** 2022-08-13

**Authors:** Julia Grube, Marius Maximilian Woitok, Antje Mohs, Stephanie Erschfeld, Celina Lynen, Christian Trautwein, Tobias Otto

**Affiliations:** Department of Internal Medicine III, University Hospital RWTH Aachen, Aachen, 52074 Germany

**Keywords:** Cancer models, Cancer genetics, Cell death

## Abstract

Ferroptosis is a novel type of programmed cell death that differs from apoptosis in that it involves iron-dependent peroxidation of membrane phospholipids. Its role in a variety of human disorders, including cancer has been hypothesized in recent years. While it may function as an endogenous tumor suppressor in a variety of cancers, its role during initiation and progression of liver cancer, particularly hepatocellular carcinoma (HCC), is yet unknown. Because HCC is most commonly found in chronically injured livers, we utilized two well-established mouse models of chronic injury-dependent HCC formation: Treatment with streptozotocin and high-fat diet as metabolic injury model, as well as treatment with diethylnitrosamine and carbon tetrachloride as toxic injury model. We used mice with hepatocyte-specific deletion of *Acsl4*, a key mediator of ferroptosis, to explore the significance of ferroptotic cell death in hepatocytes, the cell type of origin for HCC. Surprisingly, preventing ferroptotic cell death in hepatocytes by deleting *Acsl4* does not increase the formation of HCC. Furthermore, *Acsl4*-deficient livers display less fibrosis and proliferation, especially in the HCC model of toxic damage. Intriguingly, in this model, the absence of ACSL4-dependent processes such as ferroptosis significantly slow down the growth of HCC. These findings suggest that during HCC formation in a chronically injured liver, ferroptotic cell death is not an endogenous tumor-suppressive mechanism. Instead, we find that ACSL4-dependent processes have an unanticipated cancer-promoting effect during HCC formation, which is most likely due to aggravated liver damage as demonstrated by increased hepatic fibrosis. Previous studies suggested that ferroptosis might have beneficial effects for patients during HCC therapy. As a result, during HCC progression and therapy, ferroptosis may have both cancer-promoting and cancer-inhibitory effects, respectively.

## Introduction

Since the discovery of regulated cell death, most notably apoptosis, its role in numerous physiological processes and its deregulation during disease has been widely studied. Importantly, therapeutic strategies aiming at restoring apoptosis can be beneficial for cancer patients by contributing to the elimination of malignant cells [1]. While apoptosis was long regarded as the only form of regulated cell, necroptosis, pyroptosis, and ferroptosis were described as mechanistically distinct alternative cell death pathways [2].

Ferroptosis was discovered a decade ago [3], and it is defined by the peroxidation of membrane phospholipids containing polyunsaturated fatty acids (PUFAs) in the presence of iron, resulting in a necrotic-like cell death [4]. The selenoprotein glutathione peroxidase 4 (GPX4) is one of its central regulators and protects against ferroptosis and excessive accumulation of lipid peroxides using reduced glutathione [5, 6].

Other lipid peroxide repair pathways, such as GCH1-BH_4_ (ref. [7]), FSP1/AIFM2-CoQ_10_–NAD(P)H [8, 9], and DHODH-FMNH_2_-CoQ_10_ (ref. [10]), have been identified, but the GPX4 system appears to be the most essential cellular defense against ferroptotic cell death. Importantly, deletion of GPX4 in hepatocytes revealed its critical function in liver homeostasis, since it causes extensive hepatocyte ferroptosis resulting in early postnatal death in mice [11]. While the exact molecular pathways of ferroptosis are unknown, synthesis and incorporation of PUFA-containing phospholipids into cellular membranes was identified as an essential prerequisite.

Long chain acyl-CoA synthetase 4 (ACSL4) is an enzyme that catalyzes the incorporation of PUFAs into phospholipids. ACSL4 is not only enhanced during ferroptosis but also required for cells to undergo ferroptotic cell death, at least in leukemia and liver cancer cells [12, 13]. Furthermore, *ACSL4* expression was shown to be elevated in hepatocellular carcinoma (HCC) compared to normal liver tissue, suggesting that ferroptotic cell death may play a role in HCC formation [14].

HCC is the most frequent type of primary liver cancer and usually originates in a liver that has been chronically injured. In 2018, an estimated 841,000 cases of liver cancer were diagnosed worldwide, with 782,000 deaths, making liver cancer the third most common cause of death among cancers [15]. Furthermore, the number of HCC cases is increasing, particularly those that occur in the context of non-alcoholic steatohepatitis (NASH).

Several recent studies suggested that ferroptosis enhances the extent of liver inflammation and fibrosis in diet-induced NASH mouse models [16–19]. In contrast, increased expression of the ferroptosis marker *ACSL4* positively correlates with complete or partial therapy responses in human HCC patients treated with the clinically approved kinase inhibitor sorafenib [20], although it is currently under debate whether sorafenib is capable of inducing ferroptosis in HCC cells [21]. Furthermore, studies investigating different cancers, e.g., clear cell carcinoma [22], B cell lymphoma [23], and pancreatic cancer [24], suggest that ferroptosis may have an anti-cancer role. However, the involvement of ferroptosis in the development and progression of HCC has not yet been investigated.

Therefore, in this study, we aimed to examine the relevance of ferroptosis during HCC formation in NASH mouse models, comparably to human HCC development. We show that ACSL4-dependent ferroptosis in the liver does not function as an endogenous tumor suppressor but rather promotes HCC progression following chronic liver damage.

## Materials and methods

### Mouse generation and housing

To generate hepatocyte-specific *Acsl4* knockout mice (designated as *Acsl4*^Δhepa^), we combined a C57BL/6 transgenic mouse strain, Alfp-Cre [25], expressing Cre recombinase under control of the mouse albumin promoter regulatory elements and the α-fetoprotein enhancers with a mouse strain carrying floxed alleles of *Acsl4*, which originated from the European Conditional Mouse Mutagenesis Program (EUCOMM) ES clone EPD0066_2_D10. Alfp-Cre-negative littermates (*Acsl4*^f/f^) served as controls. Animals were housed under specific pathogen-free conditions in the animal facility of University Hospital RWTH Aachen at a 12 h light-dark rhythm. Mice received autoclaved food pellets and sterilized water both ad libitum.

### Mouse treatment

Induction of metabolic chronic liver injury leading to the formation of NASH and HCC was achieved by a single subcutaneous injection of 200 µg Streptozotocin (STZ; S0130; Merck Millipore, Billerica, MA, USA) into male mice two days after birth and subsequent feeding with a high-fat diet containing 60 kcal% fat lard (E15742-34; ssniff Spezialdiäten, Soest, Germany) starting at the age of five weeks until analysis of mice at the age of 12 or 20 weeks. Induction of toxic chronic liver injury leading to the formation of HCC was accomplished by a single intraperitoneal (i.p.) injection of 25 mg/kg diethylnitrosamine (DEN; N0756; Merck Millipore) into male mice at the age of 14 days, followed by weekly i.p. injections of 0.5 ml/kg CCl_4_ (289116; Merck Millipore) starting at the age of four weeks until analysis of mice at the age of 14 or 26 weeks (i.e., after 12 or 24 weeks of treatment), with the last CCl_4_ injection performed 48 h before mouse analysis. All animal experiments were approved by and carried out in accordance with the appropriate authorities for animal welfare (Authority for Environment Conservation and Consumer Protection of the State of North Rhine-Westphalia, LANUV, Recklinghausen, Germany; File Ref. 84-02.04.2017.A348).

### Cell isolation, culture, and treatment

Hepa1-6 cells were obtained from the American Type Culture Collection (ATCC), grown in DMEM supplemented with 10% fetal bovine serum and 1% penicillin/streptomycin in a humidified atmosphere containing 5% CO_2_ at 37 °C.

Primary hepatocytes were isolated from livers of 8 to 12-week-old mice with either of the two genotypes (*Acsl4*^f/f^ and *Acsl4*^Δhepa^). A laparotomy was performed following a cannulation of the *vena cava inferior*. Then the portal vein was cut, and the liver was perfused with SC1 buffer solution until the liver had blanched completely. Subsequently, the buffer was changed to SC2-containing collagenases (30 mg collagenase D and 7 mg collagenase P per 50 ml SC2 buffer). Afterwards, the liver was carefully dissected and transferred into a dish containing buffer. The digested liver was gently minced and filtered through a 70 μm cell strainer. The filtered solution was centrifuged at 50 × *g* for 2 min at 4 °C. The pellet containing hepatocytes was washed two times and checked for viability via trypan blue staining to ensure a viability of at least 80%. Primary hepatocytes were seeded in six-well plates with 250000 cells per well and cultured in HepatoZYME-SFM medium (17705021; Thermo Fisher Scientific, Waltham, MA, USA) supplemented with 1% gentamycin, 2 mM L-glutamine, and 0.02 mM phenol red in a humidified atmosphere containing 5% CO_2_ at 37 °C. 4 h after seeding, the medium was replaced. Treatment of hepatocytes was performed on the next day.

For treatment, the cells were washed carefully with PBS and 1 ml medium containing 1 or 3 µM RSL3 (S8155; Selleckchem, Houston, TX, USA), 200 nM liproxstatin-1 (SML1414; Merck Millipore), 1 µM erastin (S7242; Selleckchem), 5 µM FIN56 (S8254; Selleckchem), 10 µM FINO2 (25096; Cayman Chemical Company, Ann Arbor, MI, USA), 1000 µM arachidonic acid (10931; Merck Millipore), or DMSO (A3672,0100; AppliChem, Darmstadt, Germany) was added. For 48 h treatment, the medium was replaced after 24 h. The number of viable and dead cells was determined after 24 and 48 h by trypan blue staining.

#### Buffer composition

**Table:** 

Component	SC1	SC2
EGTA	95 mg	
Glucose	450 mg	
HEPES	1190 mg	1190 mg
KCl	200 mg	200 mg
Na_2_HPO_4_*2H_2_O	75.5 mg	75.5 mg
NaCl	4000 mg	4000 mg
NaH_2_PO_4_*H_2_O	39 mg	39 mg
NaHCO_3_	175 mg	175 mg
Phenol red	3 mg	3 mg
CaCl_2_*2H_2_O		280 mg
Total volume (in H_2_O)	500 ml	500 ml

### Tumor evaluation

Each liver was photographed with a Leica Z16 APO macroscope (Leica Microsystems, Wetzlar, Germany) from every side. Tumors on the surface of each liver with at least 2 mm in diameter were evaluated. For each of these tumors, length and width were measured, and the tumor volume was calculated as volume = length × width × width/2. For each mouse, the median tumor volume and the volume of the largest tumor were plotted. In addition, the tumor burden was calculated as sum of the volume of all tumors in an individual mouse.

### Histological staining and evaluation

For staining of formalin-fixed paraffin-embedded (FFPE) tissues, livers were fixed in 4% formaldehyde for 24 h and embedded in paraffin at the Interdisciplinary Center for Clinical Research (IZKF, University Hospital RWTH Aachen). Paraffin sections (3 µm) were cut and dried at 37 °C overnight before immunohistochemistry staining. Conventional hematoxylin and eosin (H&E) staining, Sirius Red staining, as well as Masson Trichrome staining were performed according to established protocols.

For immunohistochemistry staining of FFPE samples, liver sections were deparaffinized, boiled with 10 mM sodium citrate (pH 6.0) for 10 min, and the endogenous peroxidase activity was blocked by 3% H_2_O_2_ for 10 min. Blocking and antibody incubations were performed according to the manufacturer’s instructions using a peroxidase-conjugated polymer method (MP-7451; Vector Laboratories, Burlingame, CA, USA). The following primary antibodies were used: Cleaved caspase-3 (9661; 1:400 dilution; Cell Signaling Technology, Danvers, MA, USA), Ki67 (1220S; 1:300; Cell Signaling Technology), 4HNE (ab48506; 1:200; Abcam, Cambridge, UK), pMLKL (ab196436; 1:75; Abcam). Subsequently, antibody binding was visualized using ImmPACT DAB substrate (SK4105; Vector Laboratories) and counterstained using Mayer’s acid hematoxylin (MHS32; Merck Millipore) according to the manufacturer’s instructions. Sections were then dehydrated and mounted using Roti-Histokitt (6638.1; Carl Roth, Karlsruhe, Germany).

For immunohistochemistry/ immunofluorescence staining, liver cryosections (5 µm) were air-dried and fixed in 4% formaldehyde. After washing with PBS, unspecific binding of the antibody was blocked by incubation with blocking solution for 1 h. Sections were then incubated with a primary antibody (collagen I; 2150-1410; 1:200; Bio-Rad Laboratories, Hercules, CA, USA) overnight at 4 °C. After washing, the secondary antibody (goat anti-rabbit Alexa 546; A21085; 1:500; Thermo Fisher Scientific) was incubated for 1 h at room temperature. Subsequently, nuclei were counterstained using VECTASHIELD Antifade Mounting Medium with DAPI (H-1200; Vector Laboratories).

For detection of cell death by TUNEL (terminal deoxynucleotidyl transferase dUTP nick end labeling) staining, the In Situ Cell Death Detection Kit (11684795910; Merck Millipore) was used according to the manufacturer’s instructions to stain liver cryosections (5 µm). Subsequently, nuclei were counterstained using VECTASHIELD Antifade Mounting Medium with DAPI (H-1200; Vector Laboratories).

Imaging of histological sections was performed with an Axio Imager Z1 (Carl Zeiss, Oberkochen, Germany) for bright-field images or with an Axio Imager A2 (Carl Zeiss) for fluorescent images and processed using AxioVision LE64 version 4.9.1 software (Carl Zeiss). The investigators performing the quantification of stainings were blinded to the identity of the samples. For analysis of cleaved caspase-3, pMLKL, and TUNEL staining, quantification of positive cells was done by manual counting. For analysis of 4HNE, Sirius Red, collagen I, Masson Trichrome, and Ki67 staining, determination of positively stained area was performed using the software ImageJ (version 1.46; National Institutes of Health, USA).

### Flow cytometry

First, capillary leukocytes were removed from the liver tissue by perfusion with PBS. Then, the liver tissue was digested at 37 °C for 45 min using Collagenase type 4 (LS004189, Worthington, Lakewood, NJ, USA). Afterwards, the liver was minced through a 70 µm cell strainer, and the remaining erythrocytes were lysed using BD Pharm Lyse buffer (555899; BD, Franklin Lakes, NJ, USA). The resulting cell suspension was stained with fluorochrome-conjugated antibodies (1:300) for myeloid cells (“Mix1”) or lymphoid cells (“Mix2”) for 30 min at 4 °C.

“Mix1” contained MHCII FITC (107605; BioLegend, San Diego, CA, USA), CD11b PE (12-0112-82; Thermo Fisher Scientific), Ly6C PerCP-Cy5.5 (45-5932-82; Thermo Fisher Scientific), CD11c APC (117310; BioLegend), F4/80 PE-Cy7 (25-4801-82; Thermo Fisher Scientific), Ly6G Alexa Fluor 700 (127622; BioLegend), CD45 APC-Cy7 (557659; BD), Calibrite APC beads (340487; BD), and Hoechst 33258 as viability dye. “Mix 2” contained CD8a FITC (11-0081-85; Thermo Fisher Scientific), CD4 PE (12-0041-83; Thermo Fisher Scientific), CD19 PerCP-Cy5.5 (551001; BD), NK1.1 PE-Cy7 (25-5941-82; Thermo Fisher Scientific), CD3e APC (12-0031-81; Thermo Fisher Scientific), CD45 APC-Cy7 (557659; BD), Calibrite APC beads (340487; BD), and Hoechst 33258 as viability dye.

Sample analysis was performed using a LSRFortessa flow cytometer (BD) and FlowJo software (version 10.4.2; BD). Cells were pre-gated as Hoechst^-^ CD45^+^ to identify viable leukocytes. Total cells per liver were calculated using Calibrite APC beads for calibration.

### Lipid peroxidation assays

Lipid peroxidation was determined using BODIPY 665/676 dye, malondialdehyde (MDA) assay, or immunohistochemistry staining for 4-hydroxynonenal (4HNE; as described above). The BODIPY 665/676 dye (B3932; Thermo Fisher Scientific) was utilized as lipid peroxidation sensor in cultured cells and used according to the manufacturer’s protocol. Lipid peroxidation was then determined as mean fluorescence intensity (MFI) of BODIPY 665/676 by flow cytometry using a LSRFortessa cytometer (BD) and the PE-YG channel (excitation 561 nm, emission 582+/−7.5 nm). MDA content was measured using the Lipid Peroxidation (MDA) Assay Kit (ab118970; Abcam) following the manufacturer’s protocol.

### qRT-PCR

Total RNA was isolated from frozen liver tissue using the peqGOLD RNAPure System (30-1020; VWR International, Radnor, PA, USA) according to the manufacturer’s instructions. Reverse transcription of 2 µg total RNA into cDNA was performed with random primers using the High-Capacity cDNA Reverse Transcription Kit (4374966; Thermo Fisher Scientific). Quantitative Real-Time PCR (qRT-PCR) was performed using PowerUp SYBR Green Master Mix (A25778; Thermo Fisher Scientific) in a QuantStudio 5 Real-Time PCR System (Thermo Fisher Scientific) according to the manufacturer’s instructions. Relative mRNA expression was calculated using the 2^−ΔΔCT^ method normalizing each gene to the expression of the housekeeping genes *Gapdh* and *Actb*. Primer sequences are listed in the following table.

**Table:** 

Name	Forward primer	Reverse primer
*Acsl4*	CGCTGTTCCGGAAATCATGG	GGGGCGTCATAGCCTTTCTT
*Acta2*	TGACAGAGGCACCACTGAACC	TCCAGAGTCCAGCACAATACCAGT
*Actb*	CAGCTTCTTTGCAGCTCCTT	ATCCATGGCGAACTGGTG
*Aifm2*	GAATGTCCCCTTCATGCTGGT	TTTTTGGCGAACCCGCTCT
*Cbr3*	GGGACTTAACGTGCTGGTCA	GGGTGTTGGGTCATCCATTCT
*Ccl2*	AGCTGTAGTTTTTGTCACCAAGC	TTCCTTCTTGGGGTCAGCAC
*Ccna2*	AGCTGTCTCTTTACCCGGAG	ACGTTCACTGGCTTGTCTTCTA
*Ccnd1*	GAGCTGCTGCAAATGGAACTG	CTCATCCGCCTCTGGCATTT
*Ccne1*	GGTCTGAGTTCCAAGCCCAA	TGGTCCGTCGAGTCTCTCTC
*Col1a1*	TGTGTGCGATGACGTGCAAT	GGGTCCCTCGACTCCTAC
*Col5a2*	TGGGGACTGATGGTACACCT	GGATCACCCGATTGTCCTCG
*Dhodh*	TTCACCTCTTACCTGACAGCC	TGGAGTCCTGAAACGTAGCTC
*Gapdh*	TCAAGCTCATTTCCTGGTATGAC	CTTGCTCAGTGTCCTTGCTG
*Gch1*	TCCATTTGTAGGAAGGGTCCA	GCAATCTGTTTGGTGAGGCG
*Gpx4*	ACCCACTGTGGAAATGGATGA	CTCTATCACCTGGGGCTCCTC
*Hmox1*	GAATCGAGCAGAACCAGCCT	GCCTTCTCTGGACACCTGAC
*Il1b*	GCAGTGGTTCGAGGCCTAAT	CTCATCACTGTCAAAAGGTGGC
*Il6*	TCACTGTGCGTTGCAAACAG	GATCCGGCTGCACCATTTTT
*Mmp2*	AACGGTCGGGAATACAGCAG	GTAAACAAGGCTTCATGGGGG
*Nqo1*	TGACATCACAGGTGAGCTG	ACCACTGCAATGGGAACTG
*Ptgs2*	TGACCTGGCAGTGTATGGTG	TGTGAGTGTCGCATCAGGTC
*Slc7a11*	ATCTCCCCCAAGGGCATACT	GCATAGGACAGGGCTCCAAA
*Timp1*	GTGCACAGTGTTTCCCTGTTT	AGGACCTGATCCGTCCACAA
*Tnf*	ACTGAACTTCGGGGTGATCG	GCCATTTGGGAACTTCTCATCC
*Txn1*	ACTGCCAGGATGTTGCTG	TTCCTTGTTAGCACCGGAG

### Western blotting

For Western blot analysis, frozen liver tissue was homogenized, and whole-cell extracts were prepared using NP40 lysis buffer. Total protein lysates (40 µg) were separated by 12.5% SDS polyacrylamide gel electrophoresis (SDS-PAGE), transferred to nitrocellulose membrane, and analyzed by immunoblot with the following primary antibodies: Cyclin A (sc596; 1:1000 dilution; Santa Cruz Biotechnology, Dallas, TX, USA), PCNA (DLN-06197; 1:1000; Dianova, Hamburg, Germany), NRF2 (16396-1-AP; 1:1000; Proteintech, Rosemont, IL, USA) and GAPDH (MCA4739; 1:5000; Bio-Rad Laboratories). Secondary antibodies were HRP-linked anti-rabbit IgG (7074; 1:5000; Cell Signaling Technology) and HRP-linked anti-mouse IgG (sc-516102; 1:5000; Santa Cruz Biotechnology). The labeled proteins were visualized using enhanced chemiluminescence (RPN2232; Merck Millipore) and the resulting light emission was detected by a luminescent image analyzer ImageQuant LAS 4000 (GE Healthcare, Chicago, IL, USA).

### Blood analysis

Blood was collected from the *vena cava inferior*. In the blood serum, alanine transaminase and aspartate transaminase were measured at the Central Laboratory Facility of the University Hospital RWTH Aachen according to standard procedures.

### Statistical analysis

Data were analyzed using Prism software (version 7.01; GraphPad, San Diego, CA, USA) and are depicted as mean values with error bars indicating standard error of the mean (SEM). For each experiment, the number of animals (n) and the statistical test are indicated in the figure legend. Normal distribution within each group was verified using the D’Agostino and Pearson normality test (for *n* ≥ 7) or the Shapiro–Wilk normality test (for *n* < 7). Comparisons between two groups were analyzed by a two-tailed unpaired t-test (if both groups followed a normal distribution) or a Mann–Whitney test (if not normal-distributed). Comparisons between more than two groups (with comparison of several conditions to one control condition) were analyzed using a one-way ANOVA with Dunnett’s multiple comparisons test (if normal-distributed) or a Kruskal–Wallis test with a Dunn’s multiple comparisons test (if not normal-distributed). Differences were considered significant when p values were below 0.05. The level of significance is indicated in each figure (i.e., ****p < 0.0001, ***p < 0.001, **p < 0.01, *p < 0.05).

## Results

### Inhibition of GPX4 induces ACSL4-dependent ferroptosis in mouse hepatocytes

**Fig. 1:**
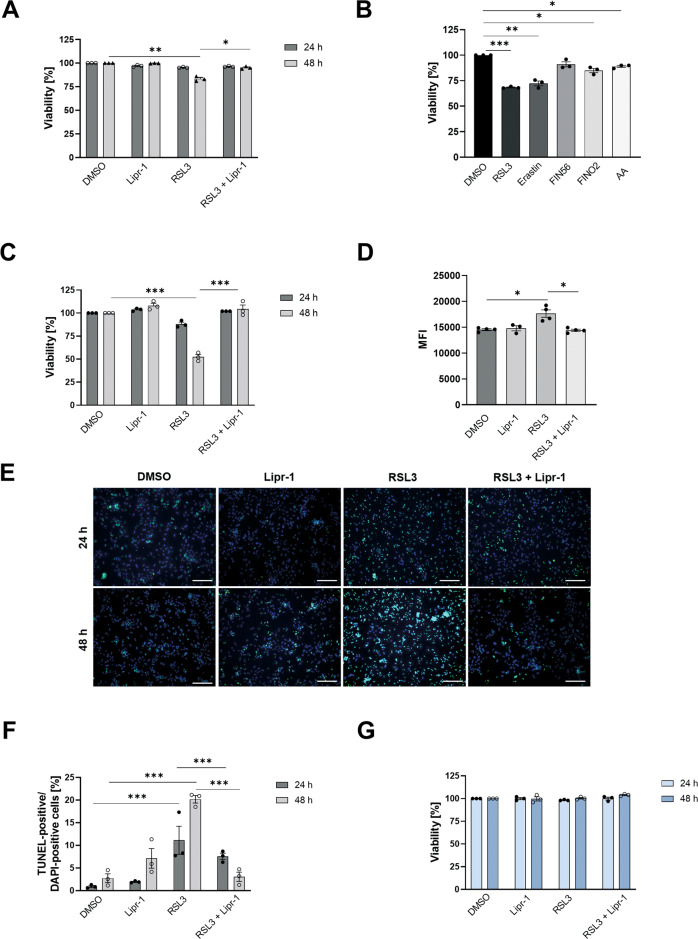
Primary Acsl4 knockout hepatocytes are resistant to ferroptosis induction. **A** Quantification of cell viability by trypan blue staining of Hepa1-6 cells after 24 or 48 h treatment with DMSO as untreated control, 3 µM RSL3, 200 nM Liproxstatin-1 (Lipr-1) or both. **B**–**G** Primary hepatocytes were isolated from 8 to 12-week-old mice. **B** Quantification of cell viability of *Acsl4*^f/f^ hepatocytes by trypan blue staining after 24 h treatment with DMSO as untreated control, 1 µM RSL3, 1 µM erastin, 5 µM FIN56, 10 µM FINO2, or 1000 µM arachidonic acid (AA). **C** Quantification of cell viability by trypan blue staining of *Acsl4*^f/f^ hepatocytes after 24 or 48 h treatment with DMSO as untreated control, 3 µM RSL3, 200 nM Lipr-1, or both. **D** *Acsl4*^f/f^ hepatocytes were treated for 24 h with DMSO as untreated control, 3 μM RSL3, 200 nM Lipr-1, or both, and stained with BODIPY 665/676 for 1 h. Lipid peroxidation was determined as mean fluorescence intensity (MFI) by flow cytometry. **E** TUNEL staining of *Acsl4*^f/f^ hepatocytes after 24 or 48 h treatment with DMSO as untreated control, 3 µM RSL3, 200 nM Lipr-1, or both. **F** Quantification of percentage of TUNEL-positive from all (DAPI-positive) cells. **G** Quantification of cell viability by trypan blue staining of *Acsl4*^∆hepa^ hepatocytes after 24 or 48 h treatment with DMSO as untreated control, 3 µM RSL3, 200 nM Lipr-1, or both. Data are expressed as mean ± SEM from one representative out of three independent experiments. *p < 0.05; **p < 0.01; ***p < 0.001; one-way analysis of variance (ANOVA) with Dunnett’s multiple comparisons test comparing each group with untreated cells for each time point.

Liver cancer cells were reported to be capable of undergoing ferroptosis [13, 26]. Hence, we evaluated the susceptibility of a mouse hepatoma cell line (Hepa1-6) to oncogenic-RAS-selective lethal compound 3 (RSL3), a ferroptosis inducer that directly suppresses the anti-ferroptotic activity of GPX4 (ref. [6, 27]). After RSL3 treatment, hepatoma cells revealed a modest reduction in viability, which was fully reversible by co-treatment with liproxstatin-1 (Fig. 1A), a radical-trapping agent that specifically suppresses ferroptosis [5].

Since cultured hepatoma cells may not faithfully recapitulate the sensitivity of liver cells to ferroptosis, we isolated primary mouse hepatocytes and treated them with mechanistically distinct inducers of ferroptosis, as well as a physiologically relevant PUFA (i.e., arachidonic acid), all of which resulted in reduced viability (Fig. 1B). Among these, RSL3 displayed the most potent cell-inhibitory effect.

Importantly, reduced viability upon RSL3 treatment was fully reversible by co-treatment with liproxstatin-1 (Fig. 1C). We also detected increased production of lipid peroxides in RSL3-treated but not in liproxstatin-1 co-treated cells (Fig. 1D), which supports the hypothesis of ferroptotic cell death. Notably, after RSL3 treatment, but not after liproxstatin-1 co-treatment, we observed an elevated percentage of TUNEL-positive cells (Fig. 1E, F), suggesting that this mode of cell death involves a measurable degree of DNA fragmentation.

Strikingly, hepatocyte-specific deletion of *Acsl4*, termed *Acsl4*^Δhepa^, completely prevented ferroptosis (Fig. 1G). These experiments demonstrate that mouse hepatocytes are susceptible to ferroptosis, which is entirely dependent on the PUFA-incorporating enzyme ACSL4.

### Abrogation of ACSL4-dependent hepatocyte ferroptosis does not enhance tumor formation in the STZ-HFD model

**Fig. 2:**
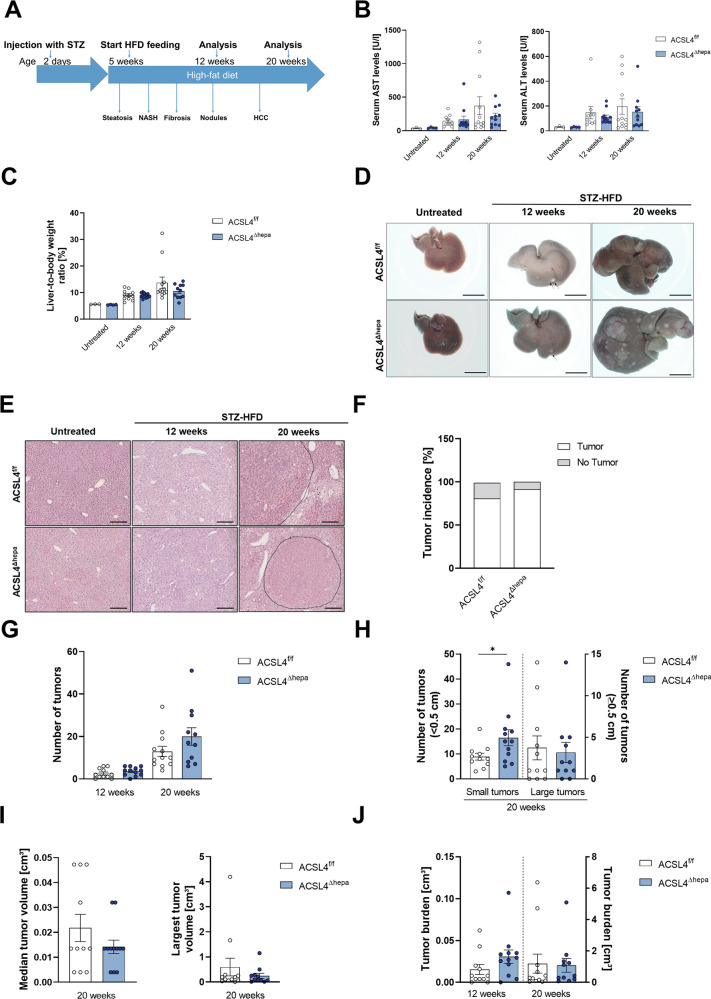
Hepatocyte-specific deletion of Acsl4 does not enhance tumor formation in STZ-HFD model. **A** Scheme of the STZ-HFD mouse model for HCC. Serum levels of aspartate transaminase (AST) and alanine transaminase (ALT) (**B**), as well as liver-to-body weight ratios (**C**) were measured in untreated, as well as *Acsl4*^f/f^ and *Acsl4*^∆hepa^ mice after 12 and 20 weeks of treatment. **D** Representative liver pictures from untreated and STZ-HFD-treated mice. Scale bars: 1 cm. **E** H&E staining of liver sections from untreated and STZ-HFD-treated mice. Scale bars: 100 µm.Tumor incidence (**F**), the total number of tumors (**G**), the number of tumors smaller than 0.5 cm and the number of tumors larger than or equal to 0.5 cm (**H**), the median tumor volume and the largest tumor volume per mouse (**I**), and the total tumor burden (**J**) were determined in *Acsl4*^f/f^ and *Acsl4*^∆hepa^ mice after 12 and 20 weeks of treatment. Data are expressed as mean ± SEM from 10 to 12 mice per group. *p < 0.05; unpaired t test comparing *Acsl4*^∆hepa^ to *Acsl4*^f/f^ mice for each time point.

To study the role of ferroptosis for liver carcinogenesis, we compared *Acsl4*^Δhepa^ mice with hepatocyte-specific *Acsl4* deletion, which completely prevents hepatocytes ferroptosis, to control mice (*Acsl4*^f/f^). These mice were investigated in the STZ-HFD model, which is a well-established model of metabolic-driven HCC development comparable to human HCC in the presence of NASH [28]. In this model, mice develop type 1 diabetes in response to early streptozotocin (STZ) injection. Additional feeding with a high-fat diet (HFD) triggers chronic liver injury and rapid development of liver steatosis and NASH, which progresses to HCC within 12–20 weeks (Fig. 2A).

We analyzed mice after 12 and 20 weeks of treatment to determine whether they developed early tumor nodules and progressed to HCC, respectively. While we observed enhanced liver injury in treated vs. untreated mice, the extent of injury was not significantly different in *Acsl4*^Δhepa^ compared to *Acsl4*^f/f^ mice (Fig. 2B). Similarly, evaluation of relative liver weight showed a time-dependent increase upon treatment with similar values between *Acsl4*^Δhepa^ and *Acsl4*^f/f^ mice (Fig. 2C).

Macroscopic evaluation of livers indicated small tumor nodules on the liver surface after twelve weeks and multiple large tumors after 20 weeks of treatment (Fig. 2D). Based on H&E-stained histological sections, these tumors were confirmed as HCC without apparent morphological differences between both genetic groups (Fig. 2E). After 20 weeks of treatment, over 80% of mice showed macroscopic tumors and differences in tumor incidence were only marginal and not statistically significant (Fig. 2F). Quantification of all macroscopically visible tumors yielded a similar number of tumors after twelve weeks of treatment (Fig. 2G). Interestingly, after 20 weeks of treatment, the tumor number seemed to be slightly elevated in *Acsl4*^Δhepa^ compared to *Acsl4*^f/f^ mice, although this difference did not reach statistical significance (Fig. 2G). Classification of these tumors into small and large tumors uncovered a significantly increased number of small tumors, but a similar number of large tumors after 20 weeks of treatment (Fig. 2H). Both the median tumor volume and the volume of the largest tumor showed a trend towards reduced tumor size without statistical significance (Fig. 2I), whereas the overall tumor burden showed a non-significant trend towards increased tumor burden after twelve weeks but no difference after 20 weeks of treatment (Fig. 2J).

These results suggest that the development of HCC in the background of NASH is not clearly affected by the lack of endogenous ACSL4-dependent ferroptotic cell death in hepatocytes. Although we observed some trends regarding tumor initiation and tumor growth, these were generally not statistically significant.

### Acsl4 deficiency in hepatocytes diminishes cell death and proliferation in the STZ-HFD tumor model

**Fig. 3:**
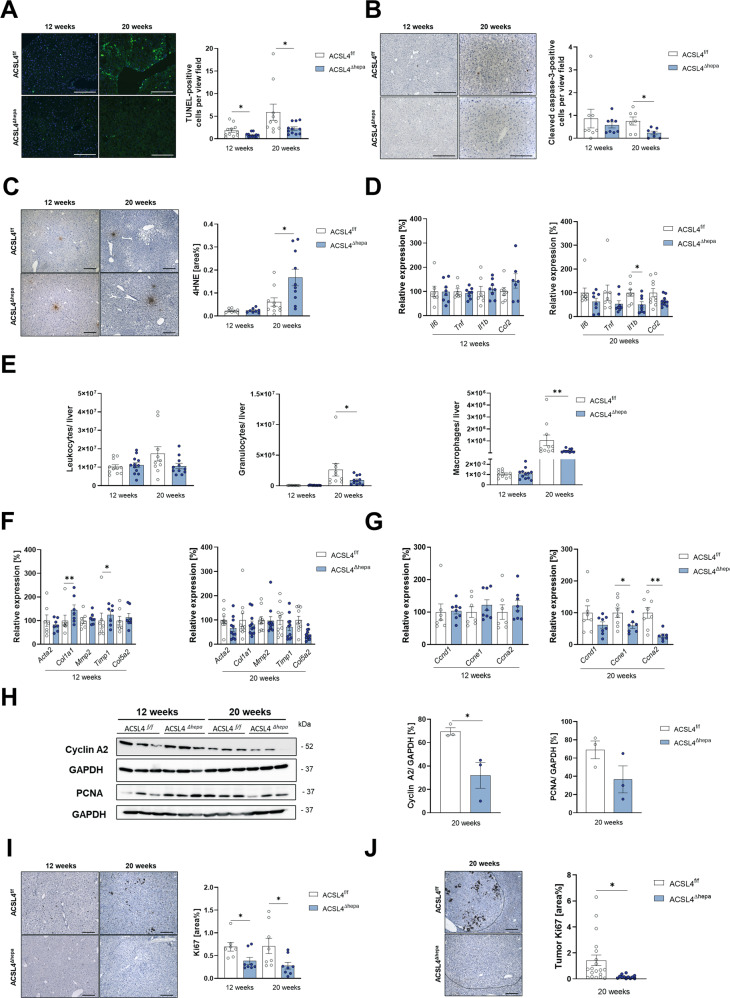
Acsl4 deficiency in hepatocytes alters cell death, inflammation, and proliferation in STZ-HFD model. TUNEL staining (**A**), cleaved caspase-3 staining (**B**), and 4-hydroxynonenal (4HNE) staining (**C**) of liver sections from *Acsl4*^f/f^ and *Acsl4*^∆hepa^ mice after 12 and 20 weeks of STZ-HFD treatment (*n* ≥ 7). Scale bars: 200 µm (**A**, **B**) or 100 µm (**C**). Quantification of TUNEL-positive cells (**A**), cleaved caspase-3-positive cells (**B**), and 4HNE-positive area (**C**) per view field. **D** Gene expression of inflammation markers (*Il6, Tnf, Il1b,* and *Ccl2*) was determined by qRT-PCR in livers and presented as relative expression compared to *Acsl4*^f/f^ mice. **E** Infiltrating leukocytes were isolated from livers. Cells were stained for CD45 to identify leukocytes and with Hoechst 33258 to exclude dead cells. Cells were stained for suitable markers and gated as neutrophil granulocytes (CD11b^+^ Ly6G^+^) or macrophages (CD11b^+^ F4/80^+^). Gene expression of fibrosis markers (*Acta2, Col1a1, Mmp2, Timp1,* and *Col5a2*) (**F**) and proliferation markers (*Ccnd1, Ccne1,* and *Ccna2*) (**G**) was determined by qRT-PCR in livers and presented as relative expression compared to *Acsl4*^f/f^ mice. **H** Immunoblot analysis of liver extracts for proliferation-related proteins (cyclin A2 and PCNA), using GAPDH as loading control. Quantification of protein levels, normalized to GAPDH. **I** Ki67 staining of liver sections (*n* ≥ 7). Scale bars: 100 µm. Quantification of Ki67-positive area was performed using ImageJ. **J** Ki67 staining of tumor areas in livers from *Acsl4*^f/f^ and *Acsl4*^∆hepa^ mice after 20 weeks of treatment (*n* ≥ 5). Quantification of Ki67-positive area within tumor areas was performed using ImageJ. Data are expressed as mean ± SEM from 10 to 12 mice per group (unless stated otherwise). *p < 0.05; **p < 0.01; ***p < 0.001; unpaired t-test comparing *Acsl4*^∆hepa^ to *Acsl4*^f/f^ mice for each time point.

To better understand the cellular and molecular changes that occur during liver carcinogenesis in the absence of ACSL4-dependent ferroptosis, we compared cell death and lipid peroxidation in livers from *Acsl4*^Δhepa^ to *Acsl4*^f/f^ mice, both treated with STZ-HFD for 12 or 20 weeks. TUNEL staining revealed a significant reduction in cell death at both time points (Fig. 3A). This might also involve apoptotic cell death as we detected a concomitant decrease in apoptosis (Fig. 3B). In contrast, we observed a mixed picture regarding necroptosis, with a drop after twelve weeks and an increase after 20 weeks of treatment, even though identifying necroptosis is technically difficult and thus less reliable (Supplementary Fig. 1A).

We predicted a lower level of lipid peroxides based on the reduced cell death and *Acsl4* deficiency in hepatocytes. However, staining for 4-hydroxynonenal, a lipid peroxidation product, revealed no change in lipid peroxidation after twelve weeks and an increase after 20 weeks of treatment (Fig. 3C), whereas no changes in lipid peroxidation were detected using the malondialdehyde assay as alternative approach (Supplementary Fig. 1B). We also confirmed that expression of *Acsl4* was drastically reduced both in tumors and surrounding liver tissue after 20 weeks of treatment (Supplementary Fig. 1C), whereas several anti-ferroptotic regulators such as *Gpx4* and *Dhodh* were elevated upon *Acsl4* deletion (Supplementary Fig. 1C–E). Concurrently, we detected increased expression of oxidative stress response genes, particularly after twelve weeks of treatment (Supplementary Fig. 1F, G). These findings suggest that *Acsl4* deficiency in hepatocytes hampers both ferroptotic and apoptotic cell death, while causing enhanced oxidative stress response and slightly elevated lipid peroxidation.

Next, we investigated three key biological mechanisms previously linked to liver cancer development: Inflammation, fibrosis, and proliferation. We observed that *Acsl4*^Δhepa^ livers had less inflammation (Fig. 3D), as well as reduced immune cell infiltration, particularly macrophages and neutrophil granulocytes, into livers after 20 weeks of treatment (Fig. 3E and Supplementary Fig. 1H–K). In addition, we detected a slight increase in liver fibrosis markers in *Acsl4*^Δhepa^ livers (Fig. 3F), albeit this did not translate into alterations in collagen fibers (Supplementary Fig. 1L).

Interestingly, we observed significantly decreased expression of several proliferation markers on mRNA and protein level (Fig. 3G–I). While these results were obtained using tumor-free liver samples or non-tumor areas, we also identified markedly reduced proliferation in tumor areas using Ki67 as well-known marker (Fig. 3J). These findings show that hepatocyte-specific deletion of *Acsl4* reduces key pro-carcinogenic processes such as inflammation and proliferation.

### Elimination of ACSL4-dependent hepatocytes ferroptosis impairs tumor progression in the DEN-CCl_4_ model

**Fig. 4:**
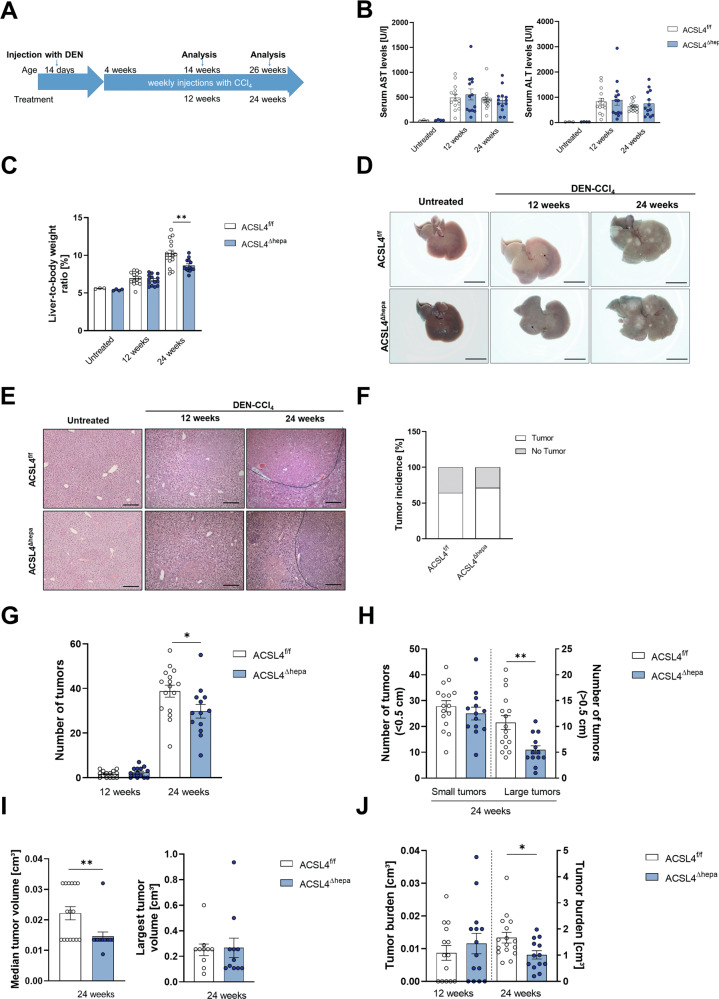
Hepatocyte-specific loss of Acsl4 reduces tumor progression in DEN-CCl_4_ model. **A** Scheme of the DEN-CCl_4_ mouse model for HCC. Serum levels of aspartate transaminase (AST) and alanine transaminase (ALT) (**B**), as well as liver-to-body weight ratios (**C**) were measured in untreated, as well as *Acsl4*^f/f^ and *Acsl4*^∆hepa^ mice after 12 and 24 weeks of DEN-CCl_4_ treatment. **D** Representative liver pictures from untreated and DEN-CCl_4_-treated mice. Scale bars: 1 cm. **E** H&E staining of liver sections from untreated and DEN-CCl_4_-treated mice. Scale bars: 100 µm. Tumor incidence (**F**), the total number of tumors (**G**), the number of tumors smaller than 0.5 cm and the number of tumors larger than or equal to 0.5 cm (**H**), the median tumor volume and the largest tumor volume per mouse (**I**), and the total tumor burden (**J**) were determined in *Acsl4*^f/f^ and *Acsl4*^∆hepa^ mice after 12 and 24 weeks of treatment. Data are expressed as mean ± SEM from 13 to 16 mice per group. *p < 0.05; **p < 0.01; unpaired t test comparing *Acsl4*^∆hepa^ to *Acsl4*^f/f^ mice for each time point.

To further analyze the role of ferroptosis, we used DEN-CCl_4_ as a second well-established HCC model [29]. In this model, injection of the carcinogen diethylnitrosamine (DEN) followed by weekly injections of the liver toxin carbon tetrachloride (CCl_4_) promotes substantial chronic liver injury triggering HCC formation within 12–24 weeks after treatment start (Fig. 4A). Although there were no differences between *Acsl4*^Δhepa^ and *Acsl4*^f/f^ mice, liver damage was elevated in treated vs. untreated mice (Fig. 4B). Notably, the relative liver weight was reduced in *Acsl4*^Δhepa^ compared to *Acsl4*^f/f^ mice after 24 weeks of treatment (Fig. 4C).

After 24 weeks of treatment, mice had developed multiple large tumors (Fig. 4D). These tumors were histologically verified as HCC with no obvious morphological changes (Fig. 4E). Although there were no differences in the tumor incidence (Fig. 4F), we observed a significant reduction in the number of macroscopically detectable tumors after 24 weeks of treatment (Fig. 4G).

In particular, we detected only half as many large tumors after 24 weeks of treatment, while the number of small tumors was unchanged (Fig. 4H). In line with this, we observed a considerable reduction in median tumor volume after 24 weeks of treatment, although the volume of the largest tumor per animal remained similar (Fig. 4I). Consequently, after 20 weeks of treatment, the overall tumor burden was significantly lower in *Acsl4*^Δhepa^ livers compared to *Acsl4*^f/f^ livers (Fig. 4J).

These findings suggest that removing ACSL4-dependent ferroptosis in hepatocytes has no effect on tumor initiation but significantly and markedly impairs tumor growth and progression in an HCC model driven by prolonged toxic liver injury.

### Acsl4 deletion in hepatocytes reduces fibrosis in the DEN-CCl_4_ model

**Fig. 5:**
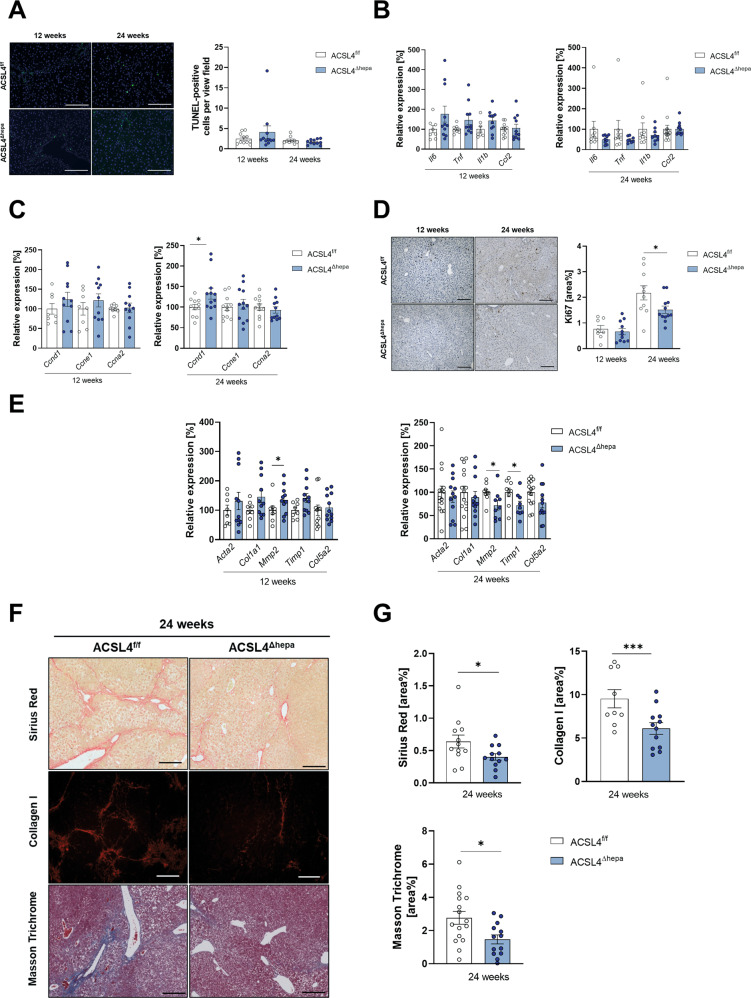
Acsl4 deficiency in hepatocytes decreases liver fibrogenesis in DEN-CCl_4_ model. **A** TUNEL staining of liver sections from *Acsl4*^f/f^ and *Acsl4*^∆hepa^ mice after 12 and 24 weeks of DEN-CCl_4_ treatment (*n* ≥ 9). Scale bars: 200 µm. Quantification of TUNEL-positive cells per view field. **B**, **C** Gene expression of inflammation markers (*Il6, Tnf, Il1b, Ccl2*) (**B**) and proliferation markers (*Ccnd1, Ccne1,* and *Ccna2*) (**C**) was determined by qRT-PCR in livers and presented as relative expression compared to *Acsl4*^f/f^ mice. **D** Ki67 staining of liver sections (*n* ≥ 8). Scale bars: 100 µm. Quantification of Ki67-positive area was performed using ImageJ. **E** Gene expression of fibrosis markers (*Acta2, Col1a1, Mmp2, Timp1,* and *Col5a2*) was determined by qRT-PCR in livers and presented as relative expression compared to *Acsl4*^f/f^ mice. **F** Sirius Red, Collagen I, and Masson Trichrome staining of liver sections (*n* ≥ 9). Scale bars: 100 µm. **G** Quantification of Sirius Red-, Collagen I-, and Masson Trichrome-positive area was performed using ImageJ. Data are expressed as mean ± SEM from 13 to 16 mice per group (unless stated otherwise). *p < 0.05; **p < 0.01; ***p < 0.001; unpaired t test comparing *Acsl4*^∆hepa^ to *Acsl4*^f/f^ mice for each time point.

Changes in cellular functions such as cell death and lipid peroxidation might explain differences in tumor progression between *Acsl4*^Δhepa^ and *Acsl4*^f/f^ mice. In contrast to the STZ-HFD model, in mice treated with DEN-CCl_4_ for 12 or 24 weeks, we observed no significant differences in the quantity of cell death and apoptotic cell death (Fig. 5A and Supplementary Fig. 2A), the amount of lipid peroxidation (Supplementary Fig. 2B, C), and the expression of oxidative stress response genes (Supplementary Fig. 2D, E). Similar to the STZ-HFD model, we confirmed drastically reduced expression of *Acsl4* (Supplementary Fig. 2E), while increased expression of anti-ferroptotic regulators upon *Acsl4* deletion was less apparent (Supplementary Fig. 2F–H).

Next, we investigated several tumor-promoting mechanisms that were previously linked to the DEN-CCl_4_ model of HCC, i.e., inflammation, proliferation, and fibrosis. Analysis of multiple inflammation indicators showed just minor changes without statistical significance (Fig. 5B). Similarly, we detected no differences in immune cell infiltration into livers (Supplementary Fig. 2I–L).

We then analyzed the amount of proliferation in tumor-free liver samples. Despite an increase in cyclin D1 expression in *Acsl4*^Δhepa^ mice after 24 weeks (Fig. 5C), we observed a significant decrease in proliferation after 24 weeks as evidenced by Ki67 (Fig. 5D). On the other hand, we were unable to detect any alteration in tumor cell proliferation (Supplementary Fig. 2M).

Importantly, even though only a few fibrosis markers showed lower mRNA expression (Fig. 5E), we observed a significant and substantial decrease in liver fibrosis after 24 weeks of treatment, as indicated by Sirius Red staining, Masson’s trichrome staining, and collagen I staining (Fig. 5F, G).

These results reveal that the previously shown reduction in tumor progression upon deletion of *Acsl4* in hepatocytes is accompanied and most likely mediated by a marked decrease in liver fibrosis in the DEN-CCl_4_ model of fibrosis-driven HCC.

## Discussion

Shortly after its discovery as a novel form of regulated cell death (ref. [3]), ferroptosis was suspected to mediate the anti-cancer effects of the multi-kinase inhibitor sorafenib, the first approved targeted therapeutic for patients with HCC [30]. Indeed, HCC cells were shown to be sensitive towards ferroptosis provoked by sorafenib or erastin [26]. At the same time, HCC cells utilize the anti-oxidative nuclear factor erythroid 2-related factor 2 (NRF2) pathway as protection from oxidative stress-mediated ferroptosis and hence, NRF2 inhibition enhances the anti-cancer activity of sorafenib in vivo [26]. Interestingly, expression of the ferroptosis mediator *ACSL4* was reported to be higher in those HCC patients that showed responses to treatment with sorafenib [31], pointing towards a potential use of *ACSL4* as predictive biomarker. However, recent results showed that many tumor cells are insensitive towards sorafenib-induced ferroptosis and hence, it is currently under debate whether sorafenib is generally applicable as ferroptosis inducer in HCC [21]. Furthermore, ferroptosis was shown to improve efficacy of cisplatin-based cancer chemotherapy in lung cancer xenograft models [32].

Although these studies provide evidence for a beneficial role of ferroptosis during HCC treatment, its relevance during HCC formation is yet unclear. Several reports investigating other malignancies point towards a tumor-suppressive role of ferroptosis, e.g., in clear cell carcinoma [33], B cell lymphoma [34], and pancreatic cancer [35]. Like apoptosis, the well-established tumor-suppressor p53 can sensitize cancer cells to ferroptosis upon increased oxidative stress and thereby inhibit tumor growth [36, 37]. Recently, one study reported that deletion of LIF receptor subunit alpha (*Lifr*) in hepatocytes not only confers resistance to ferroptosis but also promotes HCC formation, suggesting a possible link between ferroptosis and tumor suppression during liver carcinogenesis [38].

In contrast to other malignancies, HCC predominantly occurs in a background of chronic injury and metabolic stress, e.g., during NASH. These insults repeatedly trigger hepatocyte cell death, followed by inflammation, immune cell infiltration, and compensatory proliferation of hepatocytes. In addition, activation of hepatic stellate cells provokes liver fibrosis. Inflammatory signaling, compensatory proliferation, and fibrosis have been determined as key factors that drive initiation and progression of HCC.

Previous studies implicated ferroptosis to be involved in both acute and chronic liver injury. During acute liver injury, ferroptosis aggravates disease severity, and its chemical inhibition efficiently prevented liver failure and mortality in mice [39, 40].

During chronic liver injury, however, ferroptosis can have either beneficial or detrimental effects depending on the major effector cell types involved. On the one hand, chemical induction of ferroptosis in toxin- or cholestasis-induced liver fibrosis substantially reduces fibrosis development by triggering ferroptosis in hepatic stellate cells and thereby preventing their fibrosis-promoting activity [41, 42]. On the other hand, several studies have shown that ferroptosis exacerbates disease severity during chronic liver injury and that ferroptosis inhibitors play a protective role in diet-induced mouse models of NASH by reducing the extent of liver inflammation and fibrosis [16–19]. Interestingly, ferroptosis occurs at early stages of disease progression before the onset of apoptotic cell death, and its inhibition prevents subsequent liver inflammation during NASH [18]. Furthermore, chemical induction of ferroptosis enhances liver damage and inflammation in a diet-induced mouse model of NASH [17]. Notably, altering iron metabolism in hepatocytes using hepatocyte-specific deletion of the transferrin (*Trf*) gene not only increases susceptibility to ferroptosis but also to liver fibrosis [43].

While many factors have been described to mediate and contribute to ferroptosis, *ACSL4* is the only gene to date, which was shown to be essential for ferroptosis execution [44]. We herein confirm this crucial role of ACSL4 as mediator of ferroptotic cell death, specifically in mouse hepatocytes. In line with a disease-aggravating role of ferroptosis during chronic liver injury, hepatocyte-specific deletion of *Acsl4* was recently shown to reduce disease severity, in particular liver steatosis and fibrosis, in two diet-based mouse models of NASH [45]. Similarly, we observed decreased liver fibrosis in a toxic injury model of HCC (i.e., DEN-CCl_4_). Furthermore, we also detected decreased damage-associated liver inflammation and proliferation both in inflamed livers and tumor nodules using a metabolic injury model of NASH-HCC progression (i.e., STZ-HFD). Our results thus confirm earlier reports suggesting that ferroptosis contributes to several parameters of disease progression during chronic liver injury.

Intriguingly, interfering with ferroptotic cell death in hepatocytes upon *Acsl4* deficiency does not enhance HCC formation in either of the two mouse models of HCC. Of note, we observed a substantial degree of ACSL4-dependent ferroptotic and apoptotic cell death, particularly in our metabolic HCC model, which argues against the lack of ferroptosis induction during formation of NASH and HCC. However, ferroptotic cell death seems to be unable to serve as an endogenous tumor-suppressive mechanism during HCC formation in a chronically injured liver.

Furthermore, interfering with ACSL4-dependent ferroptosis clearly reduces HCC progression in the well-established toxic injury model (DEN-CCl_4_), most likely as a result of diminished fibrosis upon *Acsl4* deletion. This unexpected result indicates a tumor-promoting rather than tumor-suppressive role of ferroptosis. Interestingly, we did not observe any impact of impaired ACSL4-dependent ferroptosis on HCC formation in our metabolic NASH-HCC model (STZ-HFD). We speculate that this difference can be explained by the differences in the type of chronic liver injury between these HCC models. The toxin-induced HCC model involves a substantial degree of liver fibrosis (see Fig. 5F), which is affected by ACSL4-dependent ferroptosis and discussed as a major contributor to liver carcinogenesis in the literature. In contrast, the metabolic NASH-HCC model does not exhibit any signs of liver fibrosis (see Supplementary Fig. 1L) and is hence largely unaffected by *Acsl4* deficiency.

Notably, while ferroptosis critically depends on ACSL4, this enzyme also plays a role in lipid metabolism and might have ferroptosis-independent roles that contribute to HCC progression. For instance, previous studies suggested that ACSL4 promotes HCC formation via stabilization of the oncogenic c-Myc protein [46, 47]. While we did not detect any impact on c-Myc protein levels (data not shown), other ACSL4-dependent processes cannot be excluded. For example, ACSL4 contributes to *de novo* steroid synthesis, and chemical inhibition of ACSL4 was reported to reduce tumor growth in steroid-dependent models of breast and prostate cancer [48]. Consequently, future investigations should aim to dissect the relative contribution of ferroptosis-dependent and ferroptosis-independent roles of ACSL4 in promoting liver carcinogenesis.

In summary, we herein describe a previously unknown and unexpected pro-tumorigenic role of ACSL4-dependent ferroptosis during HCC formation in a chronically injured liver. This cancer-promoting function is most likely attributed to aggravated disease severity during chronic liver injury, as evidenced by increased liver fibrosis. While our results exclude ferroptosis as endogenous tumor-suppressive mechanism during HCC formation, several previous studies showed that ferroptosis might enhance sensitivity of liver cancer cells towards the cytotoxic effects of certain therapies.

In conclusion, ferroptotic cell death governs several aspects of chronic liver injury, HCC formation, and HCC therapy and thereby displays both cancer-promoting and cancer-inhibitory functions. These roles need to be further investigated and considered when evaluating ferroptosis as targetable mechanism during HCC therapy.

## Supplementary information

Suppl. information

Suppl. Figure 1

Suppl. Figure 2

Suppl. Figure 3

Author contribution form

Reproducibility checklist

## Data Availability

All data generated or analyzed during this study are included in this published article and its supplementary information files.
