# Supplementary material for: ACSL4-dependent ferroptosis does not represent a tumor-suppressive mechanism but ACSL4 rather promotes liver cancer progression
Source: Cell Death Dis. 2022 Aug 13;13(8):704. doi: 10.1038/s41419-022-05137-5 (PMC9376109; doi:10.1038/s41419-022-05137-5)
Supplement: Supplementary file 1 — Suppl. information [file 41419_2022_5137_MOESM1_ESM.docx]

**Supplementary Information**

***Supplementary Figure legends***

***Supplementary Fig. 1. Hepatocyte-specific deletion of Acsl4 enhances oxidative stress response in STZ-HFD model.***

**(A)** pMLKL staining of liver sections from *Acsl4*^f/f^ and *Acsl4*^∆hepa^ mice after 12 and 20 weeks of STZ-HFD treatment (n ≥ 7). Scale bars: 200 µm. Quantification of pMLKL-positive cells per view field. **(B)** Malondialdehyde (MDA) content of livers. **(C-E)** Gene expression of several ferroptotic regulators, i.e., *Acsl4* and *Gpx4* **(C)**, *Slc7a11* and *Aifm2* **(D)**, as well as *Gch1* and *Dhodh* **(E)**, was determined by qRT-PCR in paired samples from tumors and adjacent tumor-free livers after 20 weeks of STZ-HFD treatment (n ≥ 6). **(F)** Gene expression of oxidative stress markers (*Ptgs2, Cbr3, Hmox1, Nqo1, Txn1*) was determined by qRT-PCR in livers and presented as relative expression compared to *Acsl4*^f/f^ mice. **(G)** Immunoblot analysis of liver extracts for NRF2, using GAPDH as loading control. Quantification of NRF2 protein levels, normalized to GAPDH. **(H-K)** Infiltrating leukocytes were isolated from livers. Cells were stained for CD45 to identify leukocytes and with Hoechst 33258 to exclude dead cells. Cells were stained for suitable markers and gated as T cells (CD3^+^) (**H**), CD4^+^ T cells (CD3^+^ CD4^+^) or CD8^+^ T cells (CD3^+^ CD8^+^) **(I)**, B cells (CD19^+^), NK cells (CD3^-^ NK1.1^+^) **(J)**, or dendritic cells (CD11b^+^ CD11c^+^) **(K)**. **(L)** Sirius Red staining of liver sections. Scale bars: 100 µm. Quantification of Sirius Red-positive area was performed using ImageJ.

Data are expressed as mean ± SEM from 10 to 12 mice per group (unless stated otherwise). * p < 0.05; *** p < 0.001; unpaired t test comparing *Acsl4*^∆hepa^ to *Acsl4*^f/f^ mice for each time point.

***Supplementary Fig. 2. Acsl4 deletion in hepatocytes does not alter oxidative stress response or infiltration of immune cells in DEN-CCl_4_ model.***

**(A)** Cleaved caspase-3 staining of liver sections from *Acsl4*^f/f^ and *Acsl4*^∆hepa^ mice after 12 and 24 weeks of DEN-CCl_4_ treatment (n ≥ 8). Scale bars: 200 µm. Quantification of cleaved caspase-3-positive cells per view field. **(B)** 4-hydroxynonenal (4HNE) staining of liver sections (n ≥ 8). Scale bars: 100 µm. Quantification of 4HNE-positive area was performed using ImageJ. **(C)** Malondialdehyde (MDA) content of livers (n ≥ 8). **(D)** Gene expression of oxidative stress markers (*Ptgs2, Cbr3, Hmox1, Nqo1, Txn1*) was determined by qRT-PCR in livers and presented as relative expression compared to *Acsl4*^f/f^ mice. **(E)** Immunoblot analysis of liver extracts for NRF2, using GAPDH as loading control. Quantification of NRF2 protein levels, normalized to GAPDH. **(F-H)** Gene expression of several ferroptotic regulators, i.e., *Acsl4* and *Gpx4* **(F)**, *Slc7a11* and *Aifm2* **(G)**, as well as *Gch1* and *Dhodh* **(H)**, was determined by qRT-PCR in paired samples from tumors and adjacent tumor-free livers after 24 weeks of DEN-CCl_4_ treatment (n ≥ 6). **(I-L)** Infiltrating leukocytes were isolated from livers. Cells were stained for CD45 to identify leukocytes and with Hoechst 33258 to exclude dead cells **(I)**. Cells were stained for suitable markers and gated as T cells (CD3^+^ NK1.1^-^), CD4^+^ T cells (CD3^+^ CD4^+^) **(I)**, CD8^+^ T cells (CD3^+^ CD8^+^), B cells (CD19^+^), NK cells (CD3^-^ NK1.1^+^) **(J)**, macrophages (CD11b^+^ F4/80^+^) **(K)**, neutrophil granulocytes (CD11b^+^ Ly6G^+^), or dendritic cells (CD11b^+^ CD11c^+^) **(L)**. **(M)** Ki67 staining of tumor areas in livers from *Acsl4*^f/f^ and *Acsl4*^∆hepa^ mice after 24 weeks of treatment (n ≥ 5). Quantification of Ki67-positive area within tumor areas was performed using ImageJ.

Data are expressed as mean ± SEM from 13 to 16 mice per group (unless stated otherwise). * p < 0.05; unpaired t test comparing *Acsl4*^∆hepa^ to *Acsl4*^f/f^ mice for each time point.

***Supplementary Fig. 3. Original uncropped Western blot images.***
