# Supplementary figures and images for: ACSL4-dependent ferroptosis does not represent a tumor-suppressive mechanism but ACSL4 rather promotes liver cancer progression
Source: Cell Death Dis. 2022 Aug 13;13(8):704. doi: 10.1038/s41419-022-05137-5 (PMC9376109; doi:10.1038/s41419-022-05137-5)

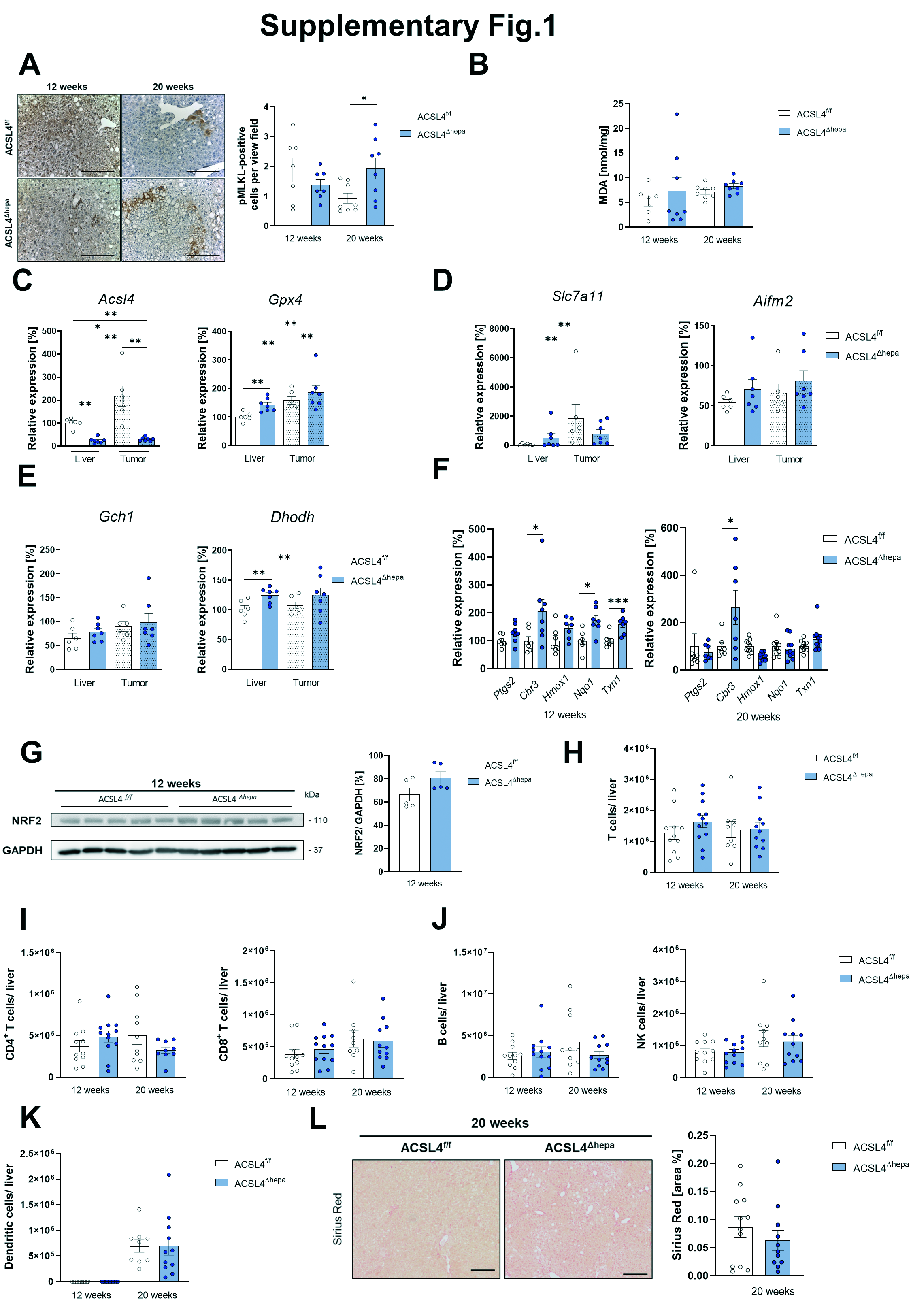

Supplement: Supplementary file 2 — Suppl. Figure 1 [file 41419_2022_5137_MOESM2_ESM.tif]

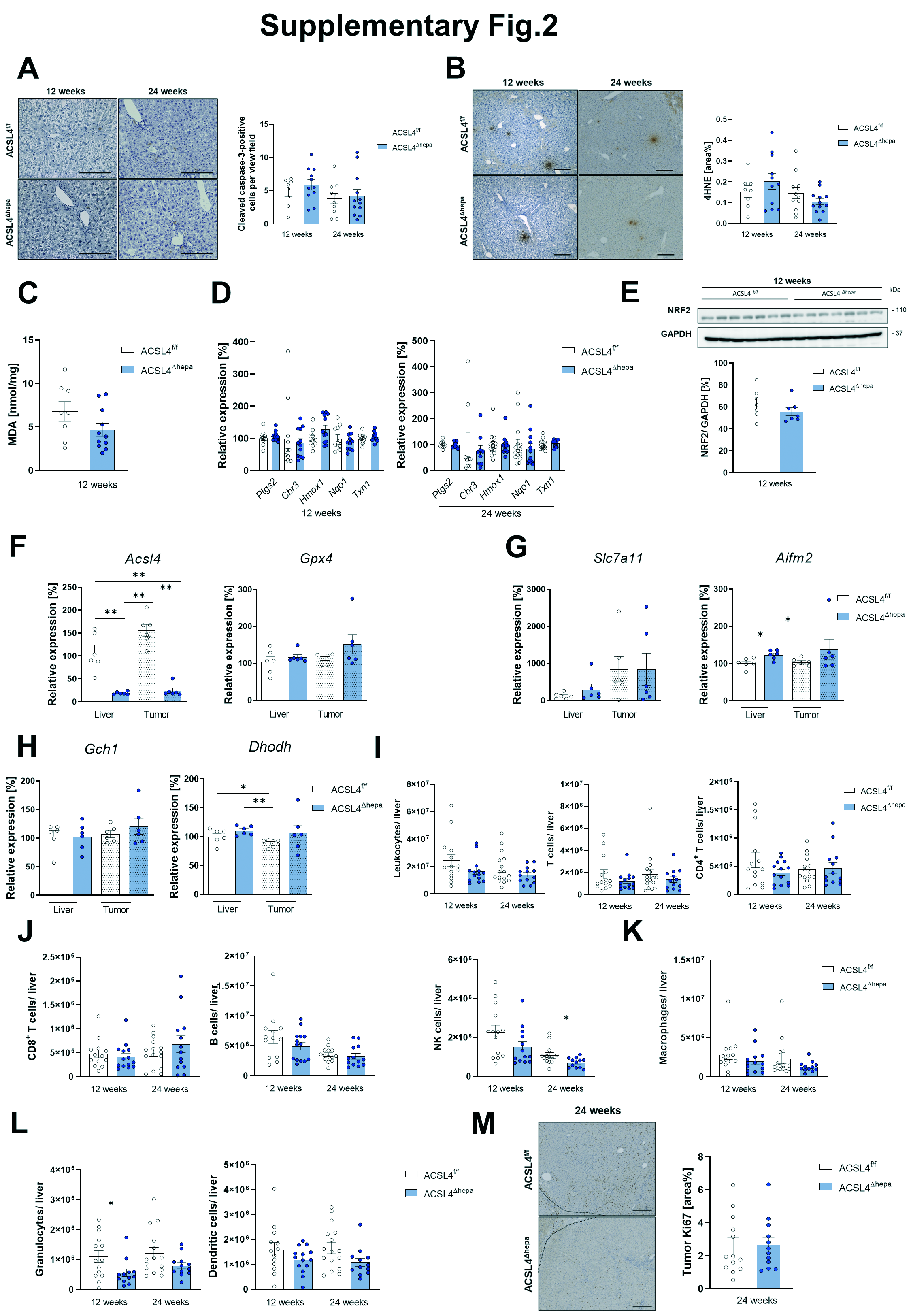

Supplement: Supplementary file 3 — Suppl. Figure 2 [file 41419_2022_5137_MOESM3_ESM.tif]
