# Supplementary material for: ACSL4-dependent ferroptosis does not represent a tumor-suppressive mechanism but ACSL4 rather promotes liver cancer progression
Source: Cell Death Dis. 2022 Aug 13;13(8):704. doi: 10.1038/s41419-022-05137-5 (PMC9376109; doi:10.1038/s41419-022-05137-5)

Supplementary Figure 3

Original Western Blots

Fig. 3H

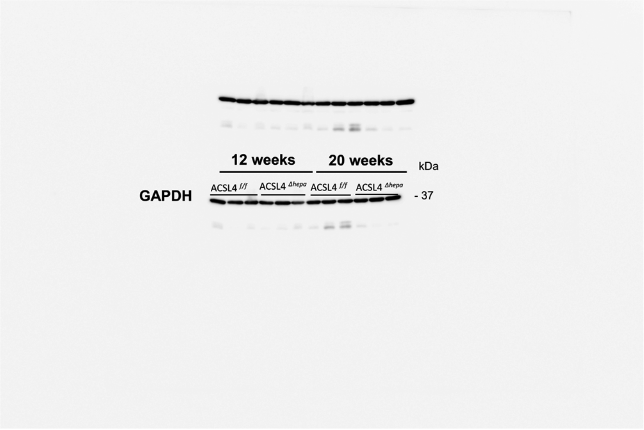

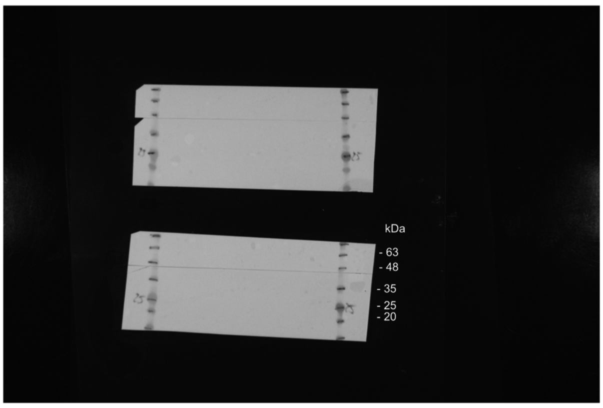

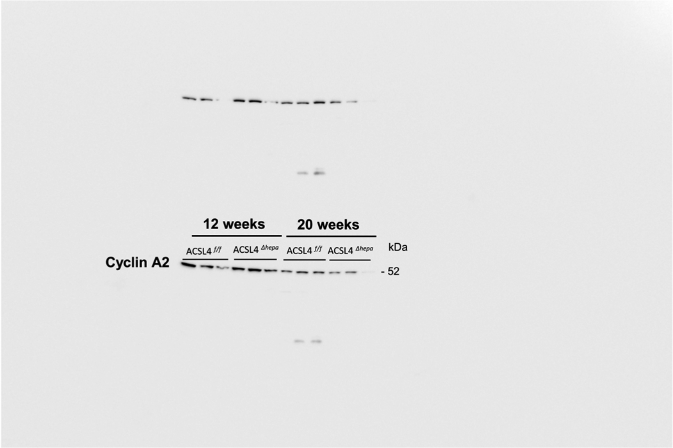

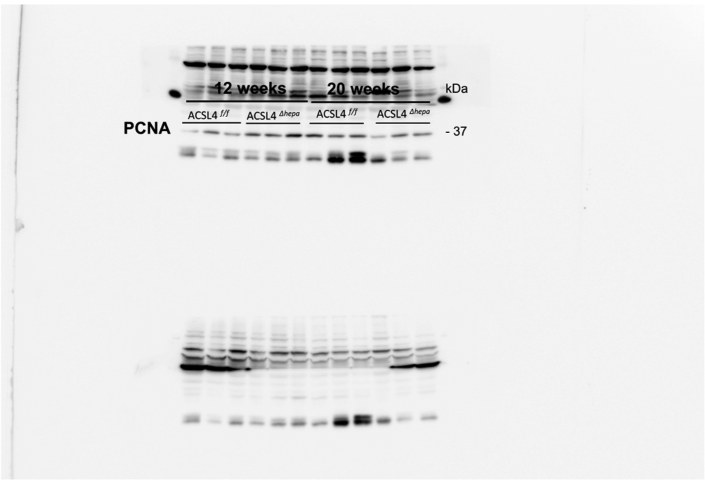

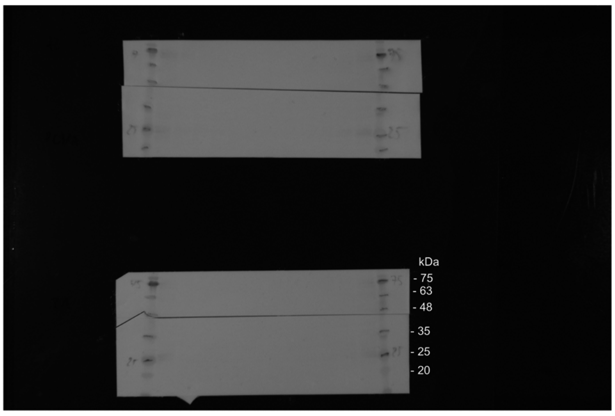

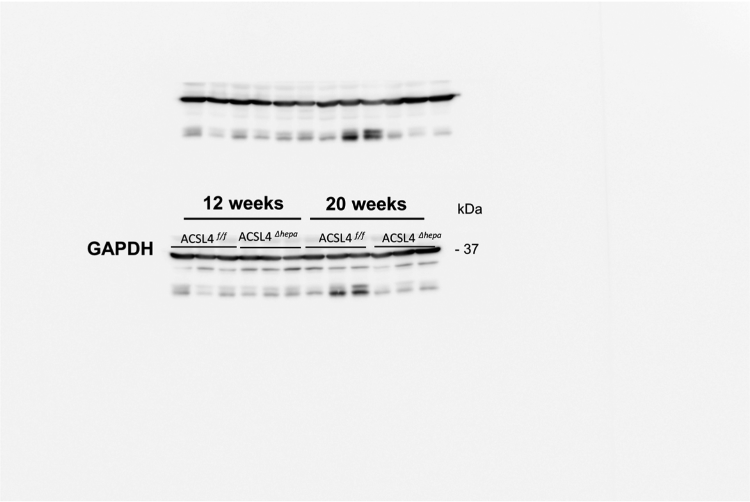

Supplementary Figure 3
Original Western Blots

Suppl Fig. 1D

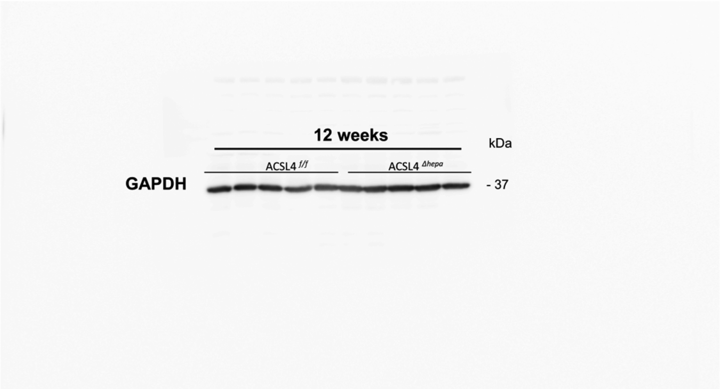

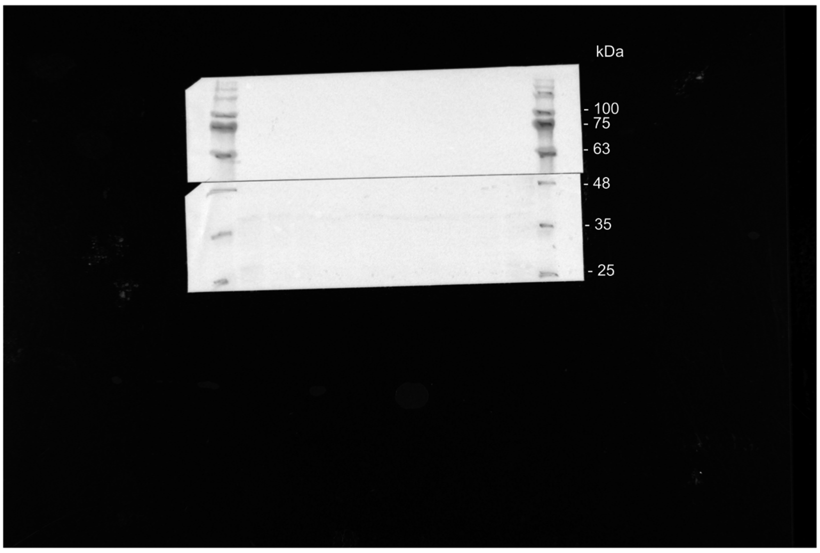

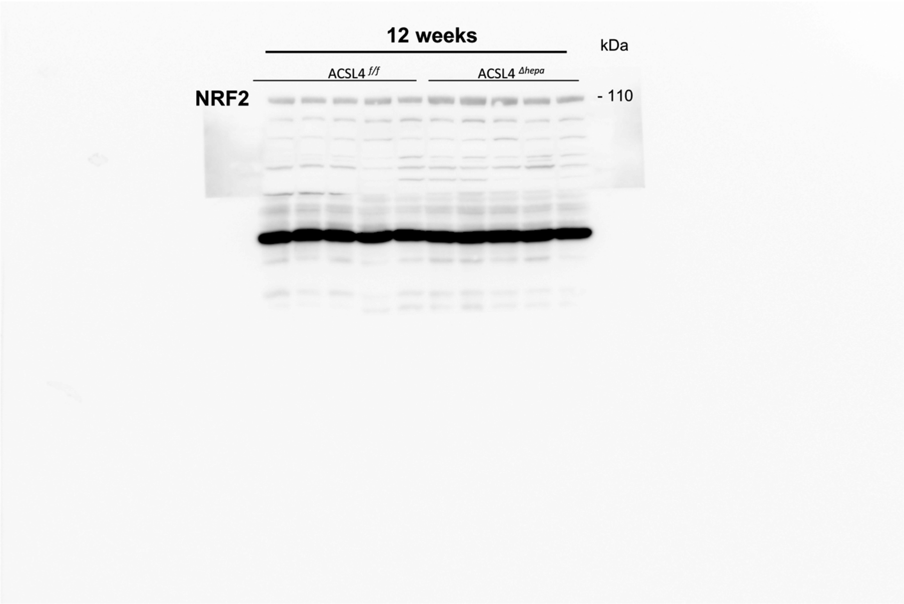

Supplementary Figure 3
Original Western Blots

Suppl Fig. 2E

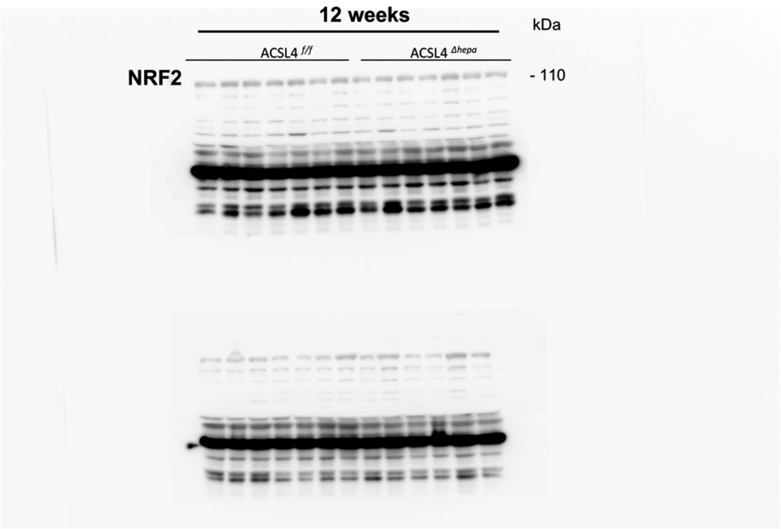

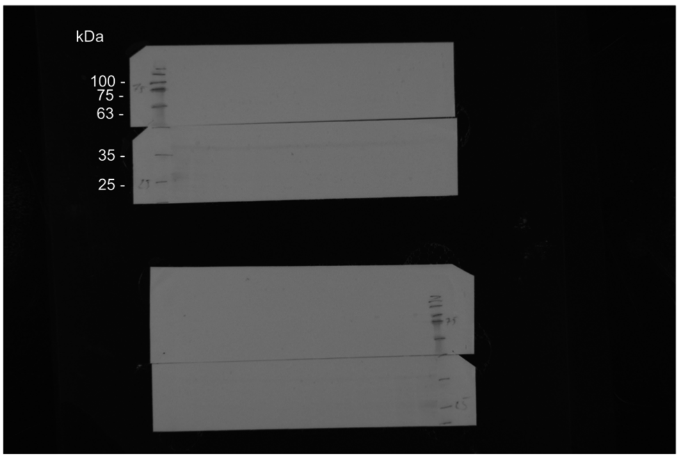

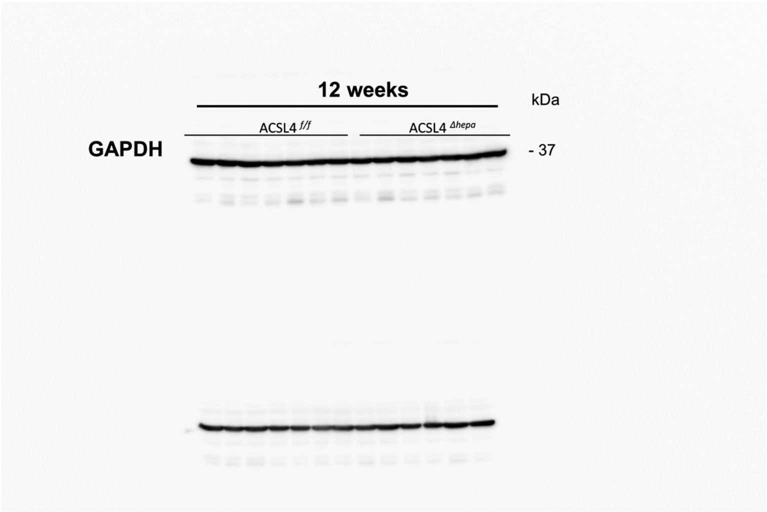

Supplement: Supplementary file 4 — Suppl. Figure 3 [file 41419_2022_5137_MOESM4_ESM.pdf]
