# Supplementary material for: ACSL4-dependent ferroptosis does not represent a tumor-suppressive mechanism but ACSL4 rather promotes liver cancer progression
Source: Cell Death Dis. 2022 Aug 13;13(8):704. doi: 10.1038/s41419-022-05137-5 (PMC9376109; doi:10.1038/s41419-022-05137-5)
Supplement: Supplementary file 5 — Author contribution form [file 41419_2022_5137_MOESM5_ESM.pdf]

DECLARATION OF CONTRIBUTIONS TO ARTICLE

ADMC

Manuscript Number:	Journal Name:	
CDDIS-22-1927-T	*Cell Death & Differentiation*	(the 'Journal')

Proposed Title of the Contribution:

ACSL4-dependent ferroptosis does not represent a tumor-suppressive mechanism but ACSL4 rather promotes liver cancer progression (the 'Contribution)

Author(s):

Julia Grube, Marius Maximilian Woitok, Antje Mohs, Stephanie Erschfeld, Celina Lynen, Christian Trautwein, and Tobias Otto (the 'Authors')

For all *CDD* articles, each person named as an author in the published version must be able to show he or she has contributed substantially to the article.

Authorship credit should be based on 1) substantial contributions to conception and design, acquisition of data, or analysis and interpretation of data; 2) drafting the article or revising it critically for important intellectual content; and 3) final approval of the version to be published. Authors should meet conditions 1, 2 and 3.

Any person who cannot be shown to have made a substantial contribution to the article cannot be listed as an author in the final version. The name of any person who is deemed to have made a minor contribution can, however, appear in the Acknowledgments section of the article.

Please complete the table below to indicate the contributions of all named authors to the manuscript.

Author Full Name:	Specification of Contribution to the Manuscript:
Julia Grube	designed the experiments, performed most of the experiments, performed data analysis, wrote the manuscript
Marius Maximilian Woitok	helped with some of the experiments (4HNE staining)
Antje Mohs	helped with some of the experiments (FACS analysis)
Stephanie Erschfeld	helped with some of the experiments (immunohistochemistry stainings, qRT-PCR)
Celina Lynen	helped with some of the experiments (mouse treatment)
Christian Trautwein	conceived and supervised the study, designed the experiments, wrote the manuscript, provided financial support
Tobias Otto	conceived and supervised the study, designed the experiments, helped with some of the experiments (mouse treatment), wrote the manuscript, provided financial support

Please complete the table below to indicate the contributions of all named authors to the figures.

Figure 1:

JG generated the data, prepared the panels, and assembled the figure.

Figure 2:

JG generated the data. TO helped with mouse treatment. JG prepared the panels and assembled the figure.

Figure 3:

JG generated the data. SE performed the immunohistochemistry stainings. MMW helped with the 4HNE staining. AM helped with the FACS analysis. JG prepared the panels and assembled the figure.

Figure 4:

JG generated the data. CL and TO helped with mouse treatment. JG prepared the panels and assembled the figure.

Figure 5:

JG generated the data. SE performed the immunohistochemistry stainings. JG prepared the panels and assembled the figure.

Figure 6:

Signed for and on behalf of the Author(s):	Print Name:	Date:
Tobias Otto	Tobias Otto	July 15, 2022
